# Conserved Gene Order and Adaptive Evolution in Mitochondrial Genomes of *Calappa* Crabs: Insights Into Ecological Specialization and Phylogenetic Utility

**DOI:** 10.1002/ece3.73282

**Published:** 2026-03-20

**Authors:** Zhengfei Wang, Huiwen Wu, Weijie Jiang, Zhiwen Xu, Yuqing Zheng, Zhixuan Wang, Jun Tang, Xin Chen

**Affiliations:** ^1^ Jiangsu Key Laboratory for Bioresources of Saline Soils, Jiangsu Synthetic Innovation Center for Coastal Bio‐Agriculture, Jiangsu Provincial Key Laboratory of Coastal Wetland Bioresources and Environmental Protection, School of Wetlands Yancheng Teachers University Yancheng Jiangsu Province China; ^2^ College of Bioscience and Biotechnology Yangzhou University Yangzhou China

**Keywords:** Calappidae, mitogenomic, phylogenetic, positive selection, rearrangement

## Abstract

Due to their maternal uniparental inheritance, structural conservation, and heterogeneous substitution rates, mitochondrial genomes are critical molecular markers for elucidating lineage diversification and reconstructing high‐resolution phylogenetic trees in marine invertebrates. Before this study, only one complete mitochondrial genome was available for the family Calappidae. We present the first complete mitochondrial genome sequences for five *Calappa* crab species. These mitogenomes exhibit characteristic features including 37 genes, a high AT nucleotide bias, and structural variation such as incomplete stop codons in certain taxa. Phylogenomic analyses confirmed the monophyly of *Calappa*. Positive selection analysis identified significant adaptive signals in energy metabolism genes (*ATP6*, *ND2*, *ND5*). Specifically, these adaptive mutations likely enhance proton transport efficiency and ATP synthesis during hypoxic burial, reflecting molecular adaptation to high‐energy‐demand environments. Notably, all examined species retain the conserved ancestral gene order of Brachyura, exhibiting remarkable stability in mitochondrial genome organization despite their ecological specialization. This conserved gene arrangement can serve as a reliable phylogenetic marker, although the molecular mechanisms underlying this stability require further investigation across additional brachyuran taxa. This study provides essential molecular resources for Calappidae systematics and Brachyuran evolutionary history, while underscoring the need to integrate future nuclear genomic data to refine higher‐level phylogenies.

## Graphical abstract

This study presents the first complete mitochondrial genomes of five *Calappa* species, filling a critical gap in genomic resources for the family Calappidae. Our analyses confirm the monophyly of Calappidae, detect positive selection in key energy metabolism genes (*ATP6*, *ND2*, *ND5*), and reveal conserved gene arrangement patterns. These findings provide fundamental molecular insights into brachyuran evolution and underscore the adaptive significance of mitogenomic architecture in marine invertebrates.

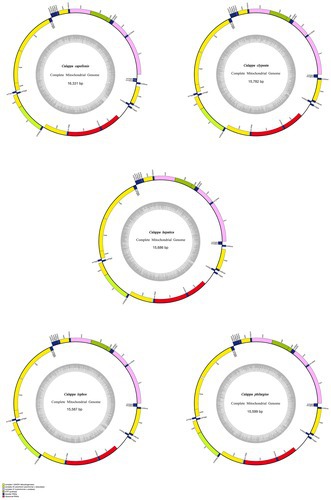

## 1 Introduction

The mitochondrial genome (mtDNA), as an extranuclear genetic material, is characterized by maternal inheritance, compact gene organization, and a high nucleotide substitution rate, rendering it particularly valuable for molecular evolution and phylogenetic studies (Boore 1999). Given these attributes, mitochondrial genomes were prioritized in this study: their accelerated evolutionary rate not only facilitates resolution of recent divergences within Brachyura but also addresses the limited discriminatory power of nuclear markers documented in earlier investigations (Ma et al. 2019). Typically, this genome exhibits a closed circular structure ranging from 14 to 20 kb in length, encompassing 13 protein–coding genes (including subunits of oxidative phosphorylation–related enzyme complexes such as *cox1–3* and *nad1–6*), 22 transfer RNA genes, 2 ribosomal RNA genes (*12S and 16S rRNA*), and a highly variable AT– rich regulatory region (Bernt, Bleidorn, et al. 2013; Bernt, Braband, et al. 2013). Compared to the nuclear genome, the mitochondrial genome offered advantages such as smaller molecular size, conserved gene composition, and absence of recombination interference, effectively circumventing the impact of nuclear pseudogenes on phylogenetic inferences. These features make it a high–resolution marker for species identification and evolutionary analyses (Gissi et al. 2008). The rapid development of next‐generation sequencing technologies, capable of handling massive molecular data, has facilitated the use of mitochondrial genomes in delivering key insights for species evolution and phylogenetic studies (Tan et al. 2018; Ruan et al. 2020; Yang et al. 2021).

The infraorder Brachyura represents a highly diverse group within the Crustacea, with over 7520 described extant species classified into approximately 100 families. Their habitats span marine, estuarine, freshwater, and terrestrial environments, playing crucial roles in global ecosystems (Marin and Tiunov 2023). Traditional classification systems primarily relied on morphological characteristics such as cheliped structure and carapace morphology to delineate familial and generic units. However, recent molecular phylogenetic studies have revealed extensive polyphyly or paraphyly within traditional crustacean taxonomic groups, suggesting that morphological convergence and ecological adaptive radiation might obscure true evolutionary relationships (Tsang et al. 2014, 2022; Wolfe et al. 2024).

The family Calappidae, represented by genera such as *Calappa* and *Matuta* and commonly known as box crabs, exhibits a broad tropical to subtropical distribution. In Chinese waters, this family has been documented with 6 genera and 20 species, accounting for approximately two‐thirds of the total species known from the Indo‐West Pacific region (Chen 1993). Galil (1997) revised the genus *Calappa*, documenting its occurrence in the Red Sea, Somalia, South Africa, and eastward through the Indian and Pacific Oceans to Easter Island (Galil 1997); Galil and Clark (1994) reported the genera *Matuta* from the Red Sea, East Africa, Japan, Australia, and Fiji (Galil and Clark 1994). Despite such a cosmopolitan distribution, these crabs share remarkably specialized burial habits to adapt to their ecological niche (Bellwood 2002). They can burrow backward into sandy substrates within seconds and remain fully buried for extended periods, while maintaining continuous respiratory water flow with the overlying water through specialized respiratory structures such as accessory water inlets and elongated water outlets. This unique burrowing lifestyle imposes specific physiological challenges on the organism's energy metabolism and oxygen utilization efficiency, particularly during periods of complete burial under hypoxic sediments (Bellwood 2002). As the “powerhouses” of cells, mitochondria have their genomes (mtDNA) encoding key subunits of respiratory chain complexes. Its evolutionary dynamics have been shown to be closely associated with adaptations related to energy metabolism (Yang et al. 2019).

To date, the NCBI database has recorded over 500 complete mitochondrial genomes of Brachyura (https://www.ncbi.nlm.nih.gov), yet two complete mitochondrial genomes from the family Calappidae have been reported. The classification and phylogeny of Calappidae have long been characterized by persistent complexity and contention, primarily due to the strikingly conserved morphology coupled with remarkably diverse ecology across species within this family (Ewers‐Saucedo et al. 2016; Camargo 2018). Although molecular studies have incorporated multiple nuclear and mitochondrial loci for Calappidae (Tsang et al. 2014; Ewers‐Saucedo et al. 2016; Wolfe et al. 2024), the phylogenetic relationships within the family are not yet fully resolved. Previous studies have proposed a Late Cretaceous origin for the superfamily Calappoidea (Tsang et al. 2014; Wolfe et al. 2024), detailed phylogenetic investigations focusing on the internal evolutionary time frame of the family Calappidae remain limited. Meanwhile, the evolutionary timeframe within and between the Calappidae family remains uncertain, and the timing of its origin and key divergence events requires further clarification.

In this study, we sequenced and analyzed the complete mitochondrial genomes of five representative species of Calappidae (*Calappa clypeata*, *Calappa capellonis*, *Calappa hepatica*, *Calappa lophos*, and *Calappa philargius*) for the first time, and compared them with other Brachyuran mitochondrial genomes. We reconstructed a Brachyuran phylogeny using 13 PCGs from 206 species, performed selection pressure analysis, and estimated divergence times to elucidate evolutionary relationships and adaptive mechanisms. Furthermore, by estimating divergence times and integrating historical event data, we elucidated the evolutionary history of Brachyura in greater detail.

## 2 Materials and Methods

### 2.1 Samples and DNA Extraction

**FIGURE 1:**
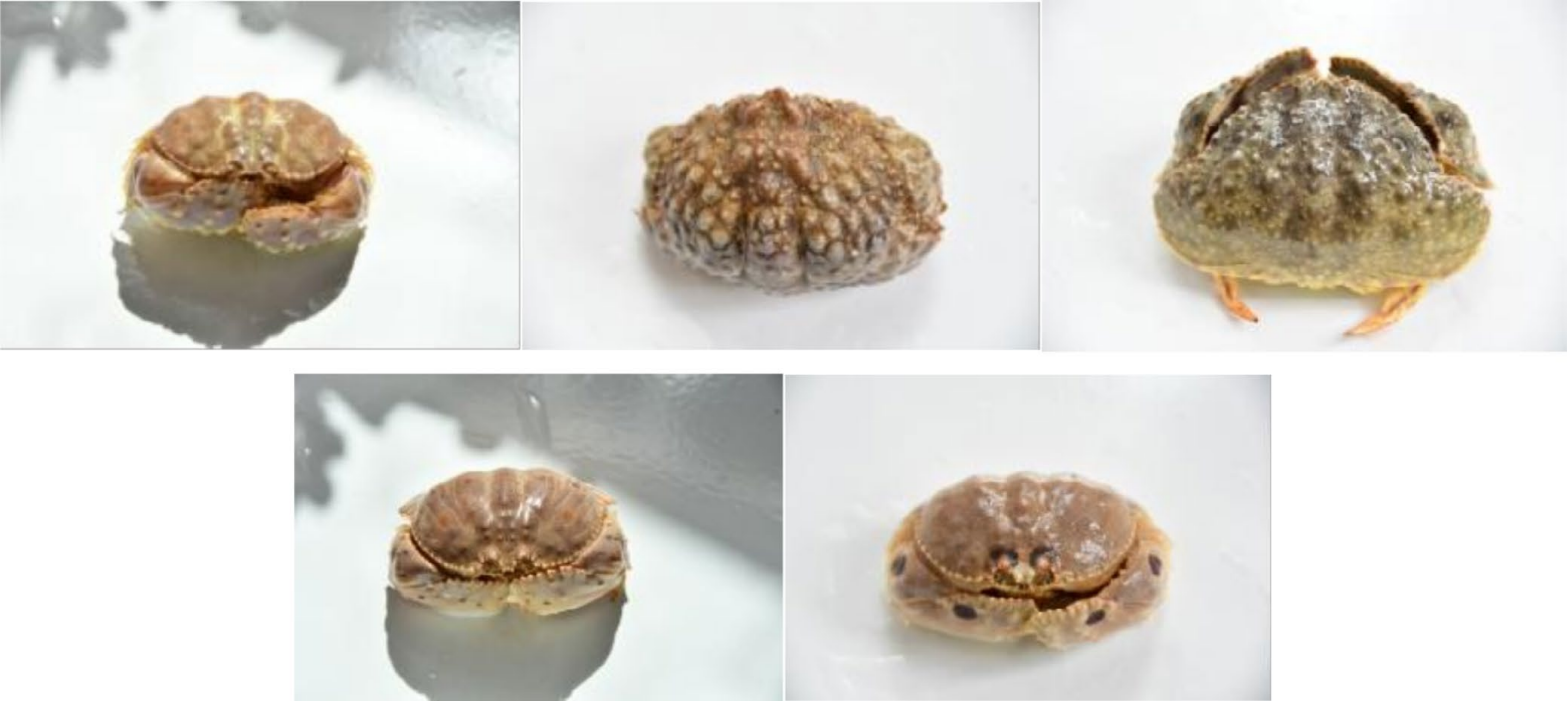
Photographs of the five study species. From left to right: *Calappa clypeata*, *Calappa capellonis*, *Calappa hepatica*, *Calappa lophos*, and *Calappa philargius*.

Five independent samples of the morphologically identified genus *Calappa* (*C*. *clypeata*, *C*. *capellonis*, *C. hepatica* , *C*. *lophos*, *C. philargius*) were collected from the waters around Hainan (the South China Sea) (Figure 1). *C. clypeata* and *C. lophos* were collected on October 9, 2022, whereas the other three species were collected on October 13, 2022. The muscle tissues were rapidly frozen in liquid nitrogen and stored at −80°C for the extraction of total genomic DNA. The Aidlab Genomic DNA Extraction Kit (Aidlab Biotech, Beijing, China) was used to extract genomic DNA from the muscle tissues, following the specific procedures outlined by the manufacturer. The quality of the extracted DNA was evaluated through electrophoresis, after which it was stored at −20°C for subsequent PCR amplification.

### 2.2 Genome Sequencing and Assembly Validation

The mitogenomes of five crabs ( *C. clypeata* , *C. capellonis*, *C. hepatica* , *C. lophos*, *C. philargius*) were sequenced by next‐generation sequencing (Illumina HiSeq 4000; Shanghai Origingene Bio‐pharm Technology Co. Ltd., China). To validate the accuracy of the species samples, the PCR was carried out in a 25 μL reaction mixture containing 12.5 μL of 2× F8 PCR MasterMix, 0.5 μL each of the universal primer *cox1* (amplifying a 711 bp fragment) and *16SrRNA* (amplifying a 568 bp fragment) (Table S7) (Sanger and Coulson 1975; Simon et al. 2006), 1.5 μL of genomic DNA template, and 10 μL of ultrapure water. Amplification was performed on an ABI9700 thermocycler under the following conditions: initial denaturation at 94°C–95°C for 4–5 min; followed by 34–35 cycles of denaturation at 94°C–95°C for 30 s, annealing at 52°C for 30 s, and extension at 72°C for 30 s; with a final extension at 72°C for 10 min. The results confirmed that the sequences were identical to those obtained through Illumina sequencing.

### 2.3 Sequence Analysis and Gene Annotation

To analyze the mitochondrial genome of invertebrates, the complete 13 PCGs were identified using tools from NCBI (https://www.ncbi.nlm.nih.gov) and the MITOS Web Server (https://mitos2.bioinf.uni‐leipzig.de/index.py). Furthermore, the potential stem–loop secondary structures within the tRNA sequences were analyzed using the MITOS Web Server and tRNAscan–SE (http://lowelab.ucsc.edu/tRNAscan‐SE/). Additionally, we calculated the nucleotide content using MEGA 7 and analyzed compositional skews with the help of the following formulas: AT–skew = (A − T)/(A + T) and GC–skew = (G − C)/(G + C). Gene maps of the five species mitogenomes were generated using the online mitochondrial visualization tools Organellar Genome DRAW and BRIG. The RNAfold WebServer (http://rna.tbi.univie.ac.at/) was utilized to predict the secondary structures of rRNA and CR, while the Tandem Repeats Finder server (http://tandem.bu.edu/trf/trf.html) was employed to detect tandem repeats within the control region.

### 2.4 Phylogenetic Analysis and Gene Rearrangement

To reconstruct the phylogenetic relationships among Brachyura crabs, we reconstructed the phylogenetic tree of 206 mitochondrial genomes, including 5 newly sequenced crab species, 184 Brachyura species, and 17 Anomura species as the outgroup (Table S1). Except the newly sequeced species, the genetic data of the other species were retrieved from GenBank. Furthermore, the nucleotide sequences of 13 PCGs were concatenated and translated into amino acid sequences using MEGA 5. Then we aligned the amino acid sequences of 13 PCGs for 206 species with MUSCLE 3.8 in MEGA 5 (Edgar 2004). The final concatenated dataset of amino acid sequences was used for further phylogenetic analyses.

The MtREV+ I + G + F model was selected as the most appropriate evolutionary models for data. The two datasets were analyzed independently using Maximum Likelihood (ML) methods available in IQ–Tree and Bayesian Inference (BI) implemented in MrBayes 3.2.6 (Huelsenbeck and Ronquist 2001; Nguyen et al. 2015). For the BI analyses, we performed two concurrent runs consisting of 10 million generations, with tree sampling occurring every 1000 generations. This setup included one cold chain and three heated chains to facilitate swapping among the Markov chain Monte Carlo (MCMC) chains. The convergence and autocorrelation were checked in the sampled parameters to ensure an effective sample size over 200, using Tracer 1.7 software. Additionally, the average standard deviation of split frequencies between the two runs was monitored to ensure it remained below 0.01. We removed the initial 25% of trees as burn–in. The Bayesian Posterior Probabilities were derived from the 50% majority rule consensus of the trees after burn–in. The final phylogenetic trees were visualized using FigTree v1.4.2 (De Bruyn et al. 2014).

In terms of gene rearrangement, MITOS and NCBI were employed to re–annotate all distinct mitogenomes for the accuracy of the data. Any discrepancies found in the mitochondrial genomes were manually corrected. To investigate the underlying evolutionary mechanisms, the CREx Web Server (http://pacosy.informatik.uni‐leipzig.de/crex) was utilized. The Common Interval Rearrangement Explorer (CREx) was applied to dynamically compute intricate rearrangement processes through mathematical modeling.

### 2.5 Selective Pressure Detection

The analysis of selective pressure was conducted by comparing the nonsynonymous/synonymous substitution ratios (*ω* = dN/dS) using the codon–based maximum likelihood (CodeML) method in the PAML4.7 package (Yang and Nielsen 2000; Welch et al. 2008). Values of *ω* < 1, *ω* = 1, and *ω* > 1 represent purifying selection, neutral evolution, and positive selection respectively. We used a branch–site model to evaluate positive selection in different branches. Employing the BEB analysis, sites under positive selection within the branch–site model were identified with a probability of 0.8. And using TreeSAAP v3.2 to detect changes in the physicochemical properties of amino acids to support the results from PAML.

### 2.6 Divergence Time Estimation

Divergence times for major lineages were estimated using BEAST v2.5.2 with uncorrelated relaxed clock and Yule tree prior models to accommodate evolutionary rate variation among branches (Drummond and Rambaut 2007). Fossil calibrations were applied based on the oldest known occurrences of clades, with 20 fossils assigned under log–normal prior distributions to reflect divergence events predating fossil records (Table S2). The dataset was partitioned to allow independent substitution rates, and analyses included preliminary runs 50 million generations. Topological constraints were refined using chronograms from initial runs. Convergence was verified in Tracer (ESS > 200), and maximum clade credibility trees were generated with TreeAnnotator. Prior settings were validated through analyses without data to prevent bias (Rambaut et al. 2018).

## 3 Results and Discussion

### 3.1 Mitogenome Organization and Base Composition

**FIGURE 2:**
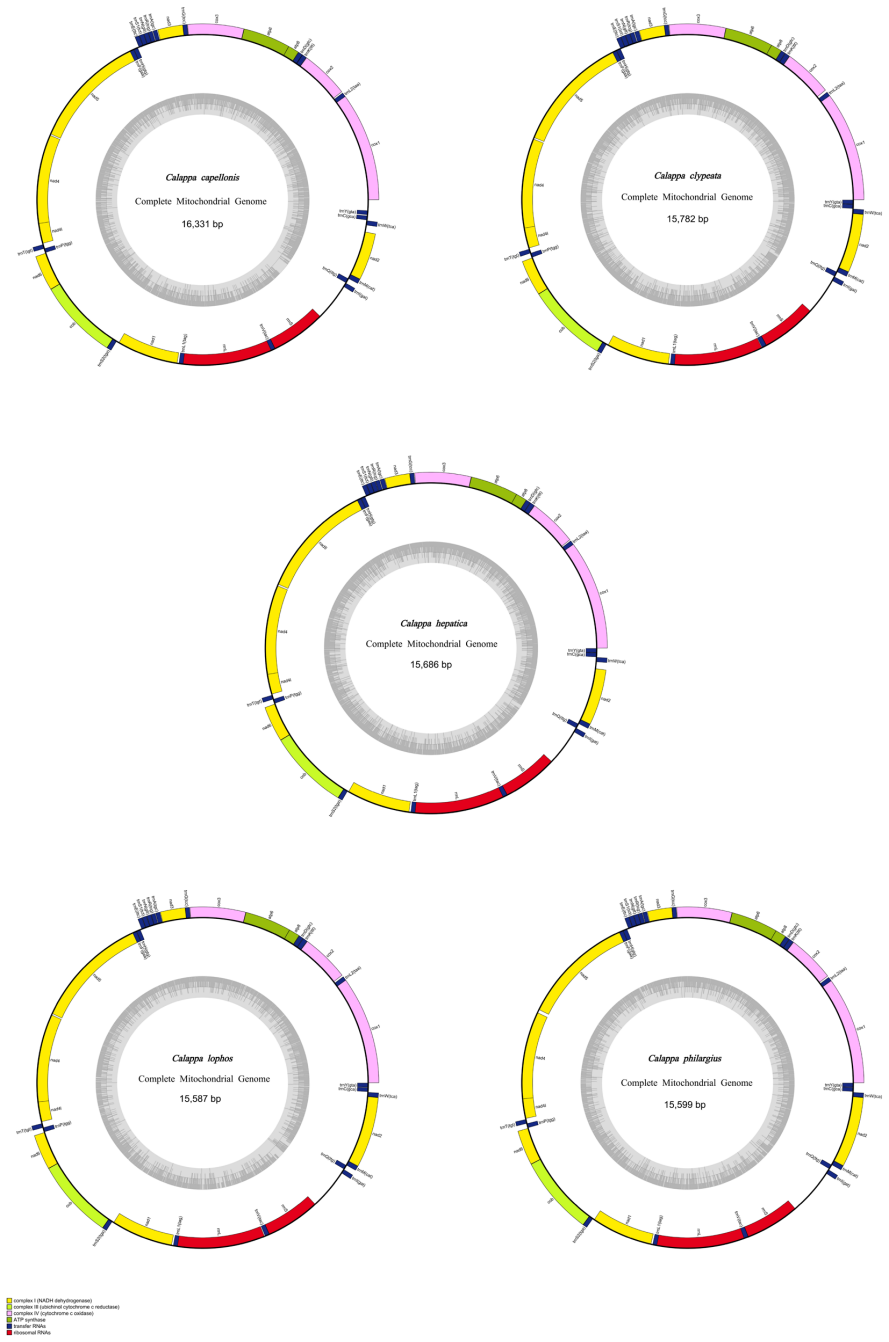
Mitochondrial genome maps of *Calappa*. Protein–coding genes are color–coded (*cox*: lavender; *nad*: yellow; *atp*: green; *cob*: kelly); rRNA genes are in red; tRNA genes are in blue. Abbreviations of protein–coding genes are: *ATP6* and *ATP8* for ATP synthase subunits 6 and 8, *cox1—3* for *cytochrome oxidase subunits 1–3*, *cob* for *cytochrome b*, *nad1—6* and *nad4l* for *NADH dehydrogenase subunits 1–6* and *4 L*, *rrnL* and *rrnS* for large and small rRNA subunits, CR for control region.

In this study, the mitochondrial genomes of five species within the genus *Calappa* (*Calappa clypeata*, *Calappa capellonis*, *Calappa hepatica* , *Calappa lophos*, *Calappa philargius*) were sequenced and fully annotated. The complete mitochondrial genomes of new species were presented with circular molecule forms ranging from 15,587 bp in *C. lophos* to 16,331 bp in *C. capellonis*, which length was similar to other mitogenomes of Brachyura from *Huananpotamon lichuanense* (15,380 bp) to *Eriocheir sinensis* (16,353 bp) (Li et al. 2016; Bai et al. 2018). The mitogenomes of five species have been deposited in GenBank under accession numbers PV686466 (*C. lophos*), PV686467 ( *C. hepatica* ), PV686468 ( *C. clypeata* ), PV686469 (*C. capellonis*), and PV686470 (*C. philargius*). All five sequenced species possess the standard set of 37 mitochondrial genes: 13 PCGs, 2 rRNAs, 22 tRNAs, and a control region (CR). The majority of the 37 genes are located on the heavy (H–) strand, except for 4 PCGs (*ND5*, *ND4*, *ND4L*, *ND1*), 8 tRNAs (*tRNA–Cys*, *Tyr*, *Gln*, *Val*, *Leu*, *Pro*, *Phe*, and *His*), and 2 rRNAs which are located on the light (L–) strand (Figure 2, Table S4). The mitochondrial genomes of the five *Calappa* species analyzed in this study exhibit a high prevalence of numerous and extended overlapping regions, a phenomenon likely driven by the combined effects of genome compactness, evolutionary events, and functional constraints. Mitochondrial genomes typically optimize genetic information storage efficiency through extreme compression of non‐coding regions, with gene overlap serving as a key strategy to enhance genetic density, a feature widely conserved during evolution (Bernt, Bleidorn, et al. 2013; Bernt, Braband, et al. 2013). Specifically, 13 overlapping regions were identified in the *Calappa* mitochondrial genomes, ranging in length from 1 to 25 bp. Notably, the longest overlapping region across all species was consistently located between *trnL1* and *rrnL* genes, maintaining a stable length of 25 bp. This conserved feature may indicate a specialized functional role in gene expression regulation or genomic structural stability. The longest intergenic spacer in *C. capellonis* (165 bp) is significantly longer than those in all other species. With the exception of *C. hepatica* and *C*. *capellonis*, whose longest intergenic spacers are co‐located between *cob* and *nad1*, the positions of these spacers differ among the remaining species (Table S4).

**FIGURE 3:**
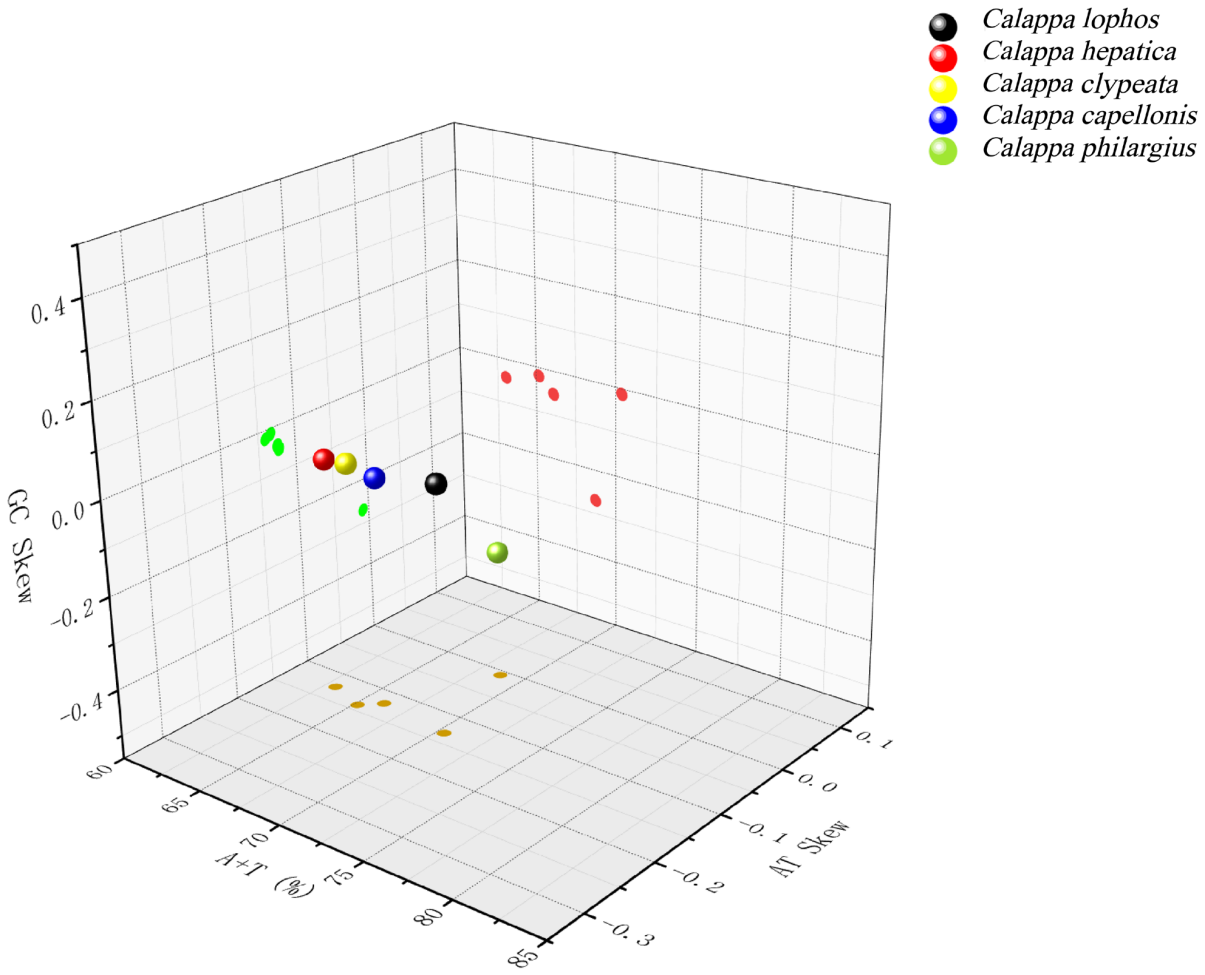
Skewed bases in the complete mitochondrial genes of five *Calappa* species.

The five *Calappa* species exhibited higher AT content, with percentages ranging from 63.01% for *C. hepatica* to a high of 70.37% for *C. lophos* in this study (Table S5, Figure 3). Both the AT–skew and GC–skew of the entire mitochondrial genome in five species were negative, indicating a prevalence of T over A and C over G (Table S5, Figure 3). The similar phenomenon also occurred in the mitochondrial genome of *Metopograpsus quadridentatus* in the Grapasidae family, with both AT skew and GC skew were negative (Wang et al. 2018).

### 3.2 PCGs and Codon Usage

The protein–coding genes (PCGs) of these five species consisted of seven NADH genes (*nad1—6* and *nad4L*), two ATP synthase genes (*atp6* and *atp8*), and four cytochrome genes (*cox1—3* and *cob*), which are commonly found in the mitochondrial genomes of invertebrate animals. Consistent with the typical mitogenome found in Brachyura, 9 PCGs were located on the heavy strand (*cox1—3*, *cob*, *atp6*, *atp8*, *nad2—3*, and *nad6*), whereas 4 PCGs were situated on the light strand (*nad1*, *nad4—5*, and *nad4L*). The majority PCGs of the five species initiated with the start codon ATN, and mostly used TAA or TAG as the stop codon. The *cox2* genes of *C. lophos*, *C. clypeata* , and *C. philargius*, along with the *nad5* genes of all five species, terminate with an incomplete stop codon T (Table S4), which is speculated to be completed through post‐ transcriptional polyadenylation to form a functional stop codon. This phenomenon has been reported multiple times in crustacean mitochondrial genomes and may be related to genome compression evolution or RNA editing mechanisms, reflecting the dynamic evolutionary characteristics of mitochondrial genomes (Hassanin et al. 2005; Gissi et al. 2008).

**FIGURE 4:**
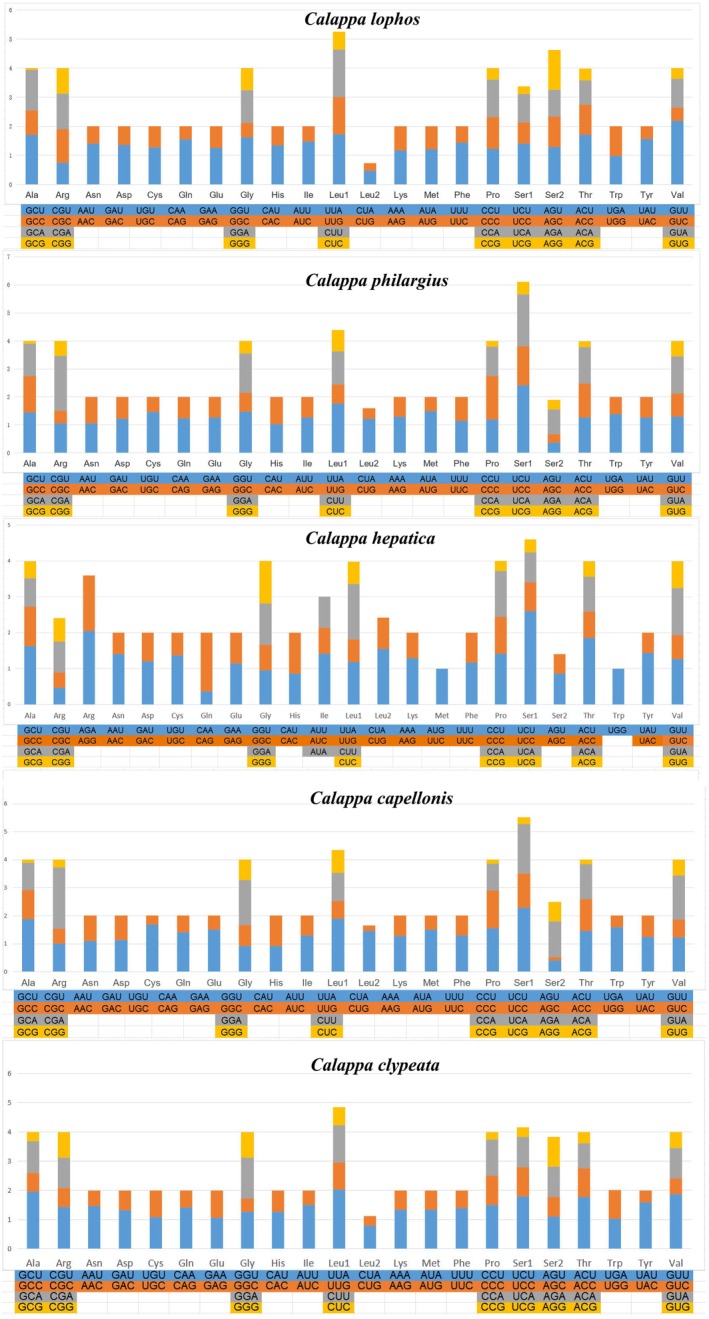
The relative synonymous codon usage (RSCU) analyses in *Calappa*.

In the 13 PCGs of *C. clypeata* , *C. capellonis*, *C. philargius* and *C. lophos*, the most frequently used amino acid was serine (Ser), accounting for 12.8%, 9.7%, 9.3%, and 16.0% respectively (Figure 3, Table S3). The most frequently used amino acid in *C. hepatica* was leucine (Leu), making up 15.2% of the usage (Figure 4, Table S3). The proportions of Isoleucine (Ile) and Phenylalanine (Phe) were also relatively high and these amino acids are all encoded by codons consisting of T or TA. This phenomenon is in accordance with the strong AT bias exhibited by the mitochondrial genome.

### 3.3 Transfer RNAs, Ribosomal RNAs, and CR

The mitogenomes of five analyzed species contained 22 tRNAs, which varied in length from 61 bp (*trnR* in five species) to 73 bp (*trnQ* in *C*. *capellonis*) (Table S4). Most tRNAs possessed the typical cloverleaf secondary structure, but a minority of tRNAs lacked the TΨC loop and DHU arm, such as *trnT* in *C*. *capellonis* lacking TΨC loop and *trnS1* in *C*. *philargius* lacking DHU arm (Figure S1). These abnormal structures are quite common in the mitochondrial genomes of metazoans, and previous studies have confirmed that they do not affect the function of *trnA* (Schneider 2011). In addition, numerous non–canonical base pairings (G–U, A–C, U–U, C–U) were identified in tRNA structures, with G–U mismatches being the most prevalent. Such mismatches are commonly observed in the tRNA molecules of various crustacean species (Wang, Tang, et al. 2020; Wang, Wang, et al. 2020), and are typically corrected through post–transcriptional tRNA modification processes (Wang, Tang, et al. 2020; Wang, Wang, et al. 2020).

The length of rRNAs in *Calappa* ranged from 2163 bp (*C. lophos*) to 2183 bp (*C*. *hepatica*). The small coding subunit (*12S rRNA*) and large coding subunit (*16S rRNA*) were all separated by the tRNA for valine (*trnV*) (Table S4). Among the five Brachyura species analyzed, the overall A + T composition of rRNA genes showed moderate AT bias, with values spanning from 68.37% ( *C. hepatica* ) to 74.42% (*C*. *lophos*) (Table S5).

The control region of the mitochondrial genome is one of the most polymorphic regions in the mitochondrial genomes of metazoans, and is widely used in phylogenetic and population genetic studies due to its high mutation rate and structural diversity (Zhang and Hewitt 1996; Saccone et al. 1999). The CR is typically located between the *tRNA—Pro* and *tRNA—Phe* genes and is the only region in the mitochondrial genome that does not encode functional proteins or RNA (Sbisa et al. 1997). Studies have shown that the CR plays a key role in regulating mitochondrial DNA replication and transcription initiation, with its core region containing conserved promoter sequences and termination–associated sequences (TAS), which are highly conserved across different species (Boore 1999; Saito et al. 2005). In addition, due to the high content of adenine and thymine in the CR, which is typically biased towards A + T nucleotides, it is also known as the AT–rich region. In the five Brachyura species examined in this study, the CR was located between *rrnS* and *trnI*. Compared to the classical CR structure, these species exhibited certain structural variations. For instance, the CR length ranged from 699 bp in *C. lophos* to 1143 bp in *C. capellonis* (Tables S4 and S5). The AT content varied from 60.04% in *C. clypeata* to 81.12% in *C. lophos*. Notably, repetitive sequences were detected in the CR of all five species (Figure S2), a feature consistent with the reported deviation from the classical compact CR structure in some crustaceans (Liu et al. 2022). These repetitive sequences may influence the structural stability and evolutionary rate of the CR, further reflecting the structural diversity of the mitochondrial control region in Brachyura species.

### 3.4 Phylogenetic Analysis

In order to further explore the phylogenetic position of Calappoidea within the Brachyura, we conducted a phylogenetic analysis based on 189 species of brachyuran crabs and 17 species of Anomura as outgroup. The two phylogenetic trees constructed (ML tree and BI tree) were largely consistent in their topological structure. Thus, only one topology with support values (BI) was displayed, including the bootstrap values for the maximum likelihood tree and the posterior probabilities supporting the Bayesian analysis.

**FIGURE 5:**
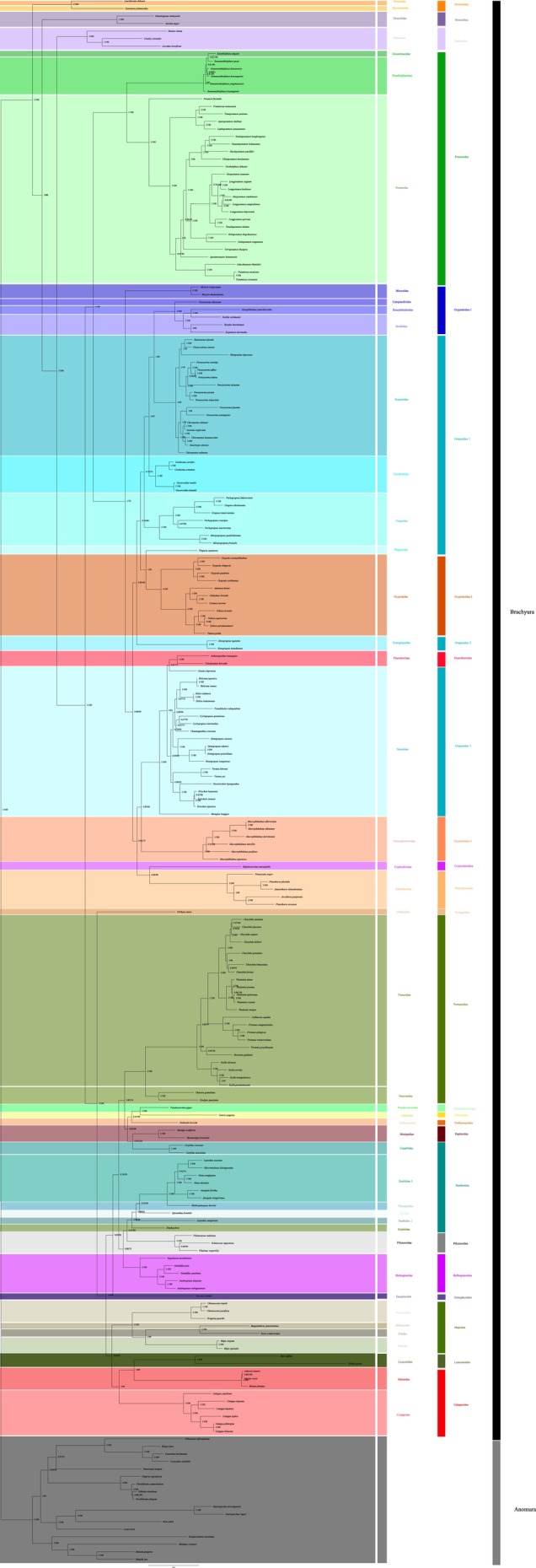
Phylogeny of 189 Brachyura species and 17 outgroups according to amino acid sequences of 13 PCGs using ML and BI analysis. Numbers on branches indicate posterior probability (BI) and bootstrap (ML).

The *Calappa* species investigated in this study ( *C. clypeata* , *C*. *capellonis*, *C*. *lophos*, *C. hepatica* , and *C*. *philargius*) exhibited a high degree of phylogenetic consistency (Figure 5). These species, together with *C. bilineata* , formed the core branch of the Calappoidea superfamily and represented a strongly supported monophyletic group (BPP/ML = 1/100). *C*. *capellonis* served as the basal lineage within the genus, while *C. clypeata* and *C. hepatica* formed a sister group (BPP/ML = 1/100), and *C*. *lophos* and *C*. *philargius*, together with *C. bilineata* , formed another highly supported clade (BPP/ML = 1/100). Phylogenetic results support the monophyly of the genus *Calappa*; however, in view of the limitations of current data, the overall monophyly of the family Calappidae still requires further verification with more extensive generic‐level data.

In this phylogenetic analysis, the majority of the 42 families formed monophyletic clades with high nodal support values. At higher taxonomic levels, most brachyuran superfamilies were monophyletic, whereas Ocypodoidea and Grapsoidea were found to be polyphyletic. These results were consistent with recent molecular phylogenetic studies (Guinot et al. 2013; Tsang et al. 2014; Wolfe et al. 2024).

The superfamilies Ocypodoidea, Grapsoidea, Cryptochiroidea, and Pinnotheroidea together form the sister group to Potamoidea. This finding is largely consistent with the results of Wang, Tang, et al. (2020) and Wang, Wang, et al. (2020), although the earlier study did not include the two superfamilies Cryptochiroidea and Pinnotheroidea (Wang, Tang, et al. 2020; Wang, Wang, et al. 2020). The family Mictyridae occupies a basal position relative to the Grapsoidea and Ocypodoidea lineages, representing the earliest diverging group, which also aligns with the results of Wang, Tang, et al. (2020) and Wang, Wang, et al. (2020). Ocypodoidea was resolved into three major clades: Ocypodidae + ((Mictyridae + Xenophthalmidae + Dotillidae + Camptandriidae) + Macrophthalmidae). Within this structure, Xenophthalmidae was consistently nested within Dotillidae. This result matches the framework for thoracotreme phylogeny proposed by Tsang et al. (2022) based on broader taxonomic sampling, thereby providing strong corroborating evidence for the systematic relationship between these two families (Tsang et al. 2022). Furthermore, our data suggest that Dotillidae is paraphyletic, which contrasts with the findings of Wang, Tang, et al. (2020) and Wang, Wang, et al. (2020), who, based on only two species, recovered the family as monophyletic (Wang, Tang, et al. 2020; Wang, Wang, et al. 2020). In our phylogenetic tree, Dotillidae (Ocypodoidea) and Xenophthalmidae (Ocypodoidea) form sister clades, and these together cluster with Camptandriidae (Ocypodoidea). These three families collectively form a group that, in turn, clusters with Sesarmidae (Grapsoidea). However, in the study by Tan et al. (2018), Sesarmidae (Grapsoidea) first clusters with Dotillidae (Ocypodoidea), and only then does this combined group form a sister relationship with Gecarcinidae (Grapsoidea) (Tan et al. 2018). In the phylogenetic tree of Zhang et al. (2023), Sesarmidae (Grapsoidea) is closely related to Gecarcinidae (Grapsoidea), while Dotillidae (Ocypodoidea) forms a sister clade with Grapsidae (Grapsoidea) (Zhang et al. 2023).

The systematic classification of the superorders Grapsoidea and Ocypodoidea has been contentious. The traditional classification system, based on morphological characteristics, assigned them to monophyletic groups. Based on 12 protein‐coding genes (PCGs), Sung et al. (2016) supported the monophyly of these two superfamilies, whereas Tsang et al. (2022), using two mitochondrial genes (*12S rRNA* and *16S rRNA*) and the nuclear histone H3 gene, demonstrated the polyphyly of Grapsoidea and Ocypodoidea. This study, along with other molecular phylogenetic studies, indicated that the superorders Grapsoidea, Ocypodoidea, and Xanthoidea exhibited significant polyphyletic characteristics, and their phylogenetic relationships need to be further resolved through the integration of multiple molecular markers. Existing research has confirmed that mitochondrial genome analysis shows important value in the phylogenetic reconstruction of crabs. However, the current sample representation is limited—some family–level units only include a single representative species, which might lead to instability in the topological structure. Therefore, future research needs to expand the sample coverage, especially by conducting complete mitochondrial genome sequencing for more representative species, in order to better explore the phylogenetic relationships ofthe short–tailed suborder.

### 3.5 Divergence Time Estimation

**FIGURE 6:**
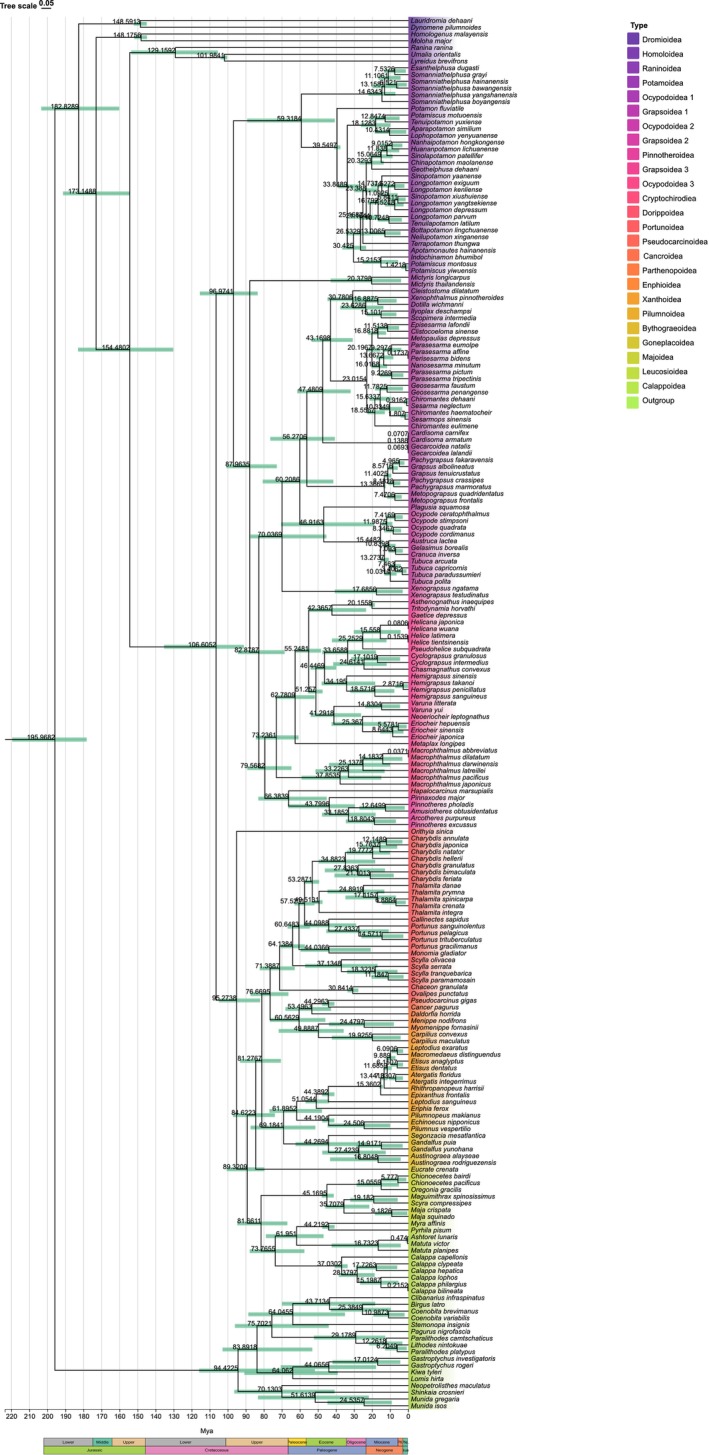
Time tree constructed using 206 species datasets, node bar indicates 95% confidence interval of the node age.

The BEAST analysis indicated that there were significant differentiation patterns within *Calappa*. Specifically, *C. capellonis* diverged first, followed by *Calappa clypeata* and *Calappa hepatica* . The divergence time between these two species was estimated to be 17.73 Ma (95% credibility interval = 6.25–29.16 Ma), suggesting that their speciation likely originated during the early Miocene (Figure 6). In contrast, the divergence time between *C. lophos*, *C*. *philargius* and *C. bilineata* was extremely recent (0.21 Ma, 95% HPD = 0.02–0.73 Ma), indicating that the latter two may have emerged as sister species through rapid speciation or microhabitat adaptation during the Pleistocene to Holocene (Figure 6). These findings are consistent with the research on recent divergence in crabs by (Wolfe et al. 2019), who noted that climate change and sea–level fluctuations may have accelerated reproductive isolation in shallow–water crabs (Wolfe et al. 2019).

Molecular clock analysis indicates that Brachyura originated in the Early Jurassic (approximately 182.83 Ma), consistent with earlier mitogenomic and morphological studies suggesting a Jurassic crown‐group origin (Tsang et al. 2014). At higher taxonomic levels, the diversification of major superfamilies exhibits distinct temporal clustering: the divergence between Grapsidoidea and Ocypodoidea occurred at approximately 66–80 Ma (Late Cretaceous to Paleogene), Portunoidea appeared at 71.39 Ma (Late Cretaceous), Potamoidea diverged at 59.32 Ma (Paleocene/Eocene boundary), and Xanthoidea split from Pilumnoidea at 61.90 Ma. These temporal nodes cluster around the Cretaceous‐Paleogene (K‐Pg) boundary, suggesting that the divergence of major brachyuran lineages was closely associated with the end‐Cretaceous mass extinction and subsequent ecological restructuring.

This temporal framework is comparable to recent large‐scale phylogenetic studies based on transcriptomes and nuclear genes. Luque et al. (2024), integrating 36 fossil calibration points, supported major familial divergences occurring between the Late Cretaceous and Paleogene (Luque et al. 2024). Pan et al. (2024), based on transcriptomic data from 56 species, similarly inferred that the diversification of major brachyuran superfamilies and families was concentrated around the Cretaceous‐Paleogene transition (Pan et al. 2024). Wolfe et al. (2024), using multi‐gene analyses, also demonstrated that splits among major lineages occurred in the Late Cretaceous to early Paleogene (approximately 66 Ma) (Wolfe et al. 2024). Although these studies, based on nuclear genomes or transcriptomes, may estimate earlier crown‐group origins, their divergence time estimates at familial and superfamilial levels (59–80 Ma) align closely with our mitogenomic inference, collectively supporting a rapid radiation of Brachyura near the K‐Pg boundary.

It should be noted that differences in evolutionary dynamics between mitochondrial and nuclear genomes may lead to systematic biases in estimating deep divergence times. Mitochondrial protein‐coding genes evolve faster and are subject to population genetic processes, potentially generating slight rate heterogeneities at deep nodes and yielding somewhat younger estimates than transcriptomic data (Tsang et al. 2014). Additionally, the selection of fossil calibrations and the application of relaxed molecular clock models may affect the resolution of Triassic‐Jurassic deep divergences. Nonetheless, our familial and superfamilial divergence estimates are consistent with the latest fossil calibration frameworks (Luque et al. 2024) and transcriptomic molecular clocks (Pan et al. 2024; Wolfe et al. 2024), confirming the diversification of major brachyuran lineages during the Late Cretaceous to early Paleogene.

### 3.6 Gene Rearrangement

In the Brachyura, mitochondrial gene arrangement is widely recognized as being subject to strong evolutionary constraints, with most families retaining the ancestral arrangement pattern (Bernt, Bleidorn, et al. 2013; Bernt, Braband, et al. 2013). The gene arrangement analysis of the Calappoidea superfamily in this study further corroborates this trend, revealing that all examined species (including five Calappidae species and three Matutidae species) conform to the ancestral gene order of the Brachyura, with no rearrangement events detected.

From an ecological perspective, Matutidae species are characterized by active swimming and predatory capabilities, whereas Calappidae are specialized for a substrate‐burrowing lifestyle (Bellwood 2002). Despite these differences, both groups exhibit high consistency in mitochondrial gene arrangement, which stands in stark contrast to the frequent rearrangement events documented within the Anomura (Tan et al. 2018). Based on the conservation observed in Calappoidea, our results suggest that the lineage to which this superfamily belongs may have inherited the shared genomic stability characteristics of the Brachyura. Consequently, its conserved gene arrangement can serve as a reliable phylogenetic marker, although the molecular mechanisms driving this stability require further investigation across additional brachyuran taxa.

### 3.7 Selective Pressure

**FIGURE 7:**
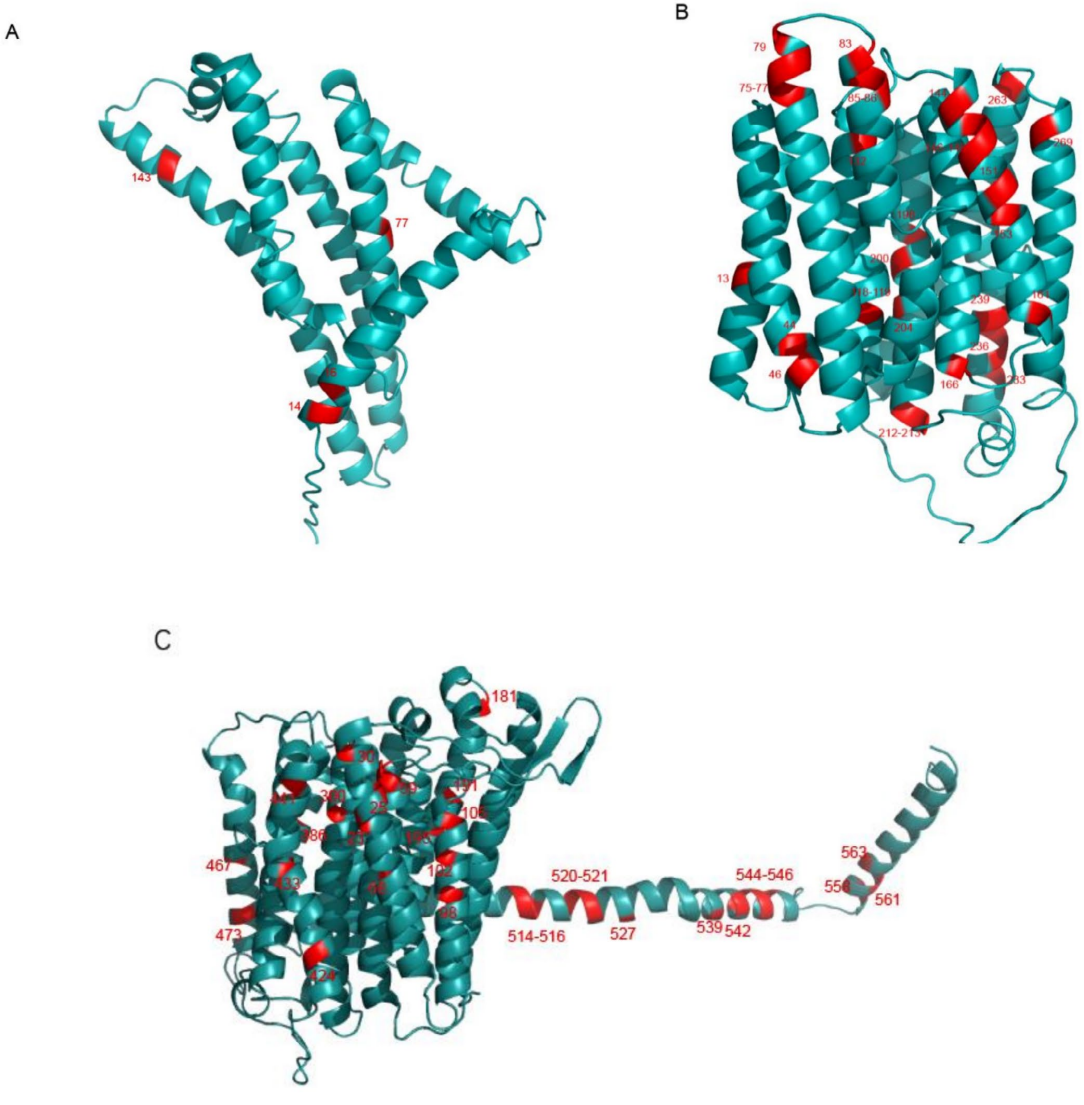
Three‐dimensional representation of *ATP6* (A), *ND2* (B) and *ND5* (C) protein, with detected positive selection sites labeled in red.

To investigate the adaptive evolution of mitochondrial genes in Brachyura crabs, we employed CodeML software and applied the branch–site model to analyze selection pressures on PCGs across 206 crab species, while determining the ratio of nonsynonymous to synonymous substitution rates (dN/dS, denoted as *ω*). We found that the *ATP6*, *ND2*, and *ND5* genes showed significant signals of adaptive evolution and had multiple positive selection sites (Figure 7, Table S6). The *ATP6* gene exhibited extremely high selection pressure in the ma model (*ω*2 = 157.543, *p* = 0.002), significantly surpassing the neutral model (ma0, *ω*2 = 1.0) (Table S6). This gene encodes a subunit of mitochondrial ATP synthase, which is directly involved in proton transmembrane transport and ATP synthesis (Ballard and Whitlock 2004). The strong positive selection observed may reflect adaptive demands for metabolic depression and energy conservation, particularly associated with hypoxia tolerance in decapod crustaceans. Similarly, significant test results for *ND2* (2ΔlnL = 5.947, *p* = 0.0147; *ω*2 = 999.0) and *ND5* (2ΔlnL = 37.394, *p* < 1e‐9; *ω*2 = 11.923) further corroborate the presence of positive selection (Table S6). Elevated *ω* values suggest that adaptive functional divergence at specific loci in these genes may arise from nonsynonymous mutations (Kosakovsky et al. 2011). The high *ω* value (*ω*2 = 999.0) of *ND2* suggests that nonsynonymous mutations at these sites may enhance mitochondrial respiratory efficiency, facilitating ATP production for the energetically intensive processes of sediment burial and durophagous feeding characteristic of Calappidae species (McGaw 2005; Seed and Hughes 1995). Furthermore, we found that the vast majority of positively selected sites are clustered in transmembrane domains, which is functionally significant as these regions are critical for proton translocation and the structural integrity of respiratory chain complexes (Dhar et al. 2021).

Notably, the adaptive evolutionary signal in the *ND5* gene was particularly significant (*ω*2 = 11.923, 2ΔlnL = 37.394, *p* = 9.65e–10), and multiple high–confidence positively selected sites were detected (Table S6). *ND5* is a core subunit of mitochondrial respiratory chain Complex I, and its function is closely related to the efficiency of oxidative phosphorylation. The positive selection likely reflects dynamic regulation of energy metabolism by this gene in response to varying environmental pressures (Ballard and Whitlock 2004). In contrast, the *ω*2 value of the *COX1* gene (1.25) was slightly higher than the neutral threshold (*ω* = 1), but the statistical test was not significant (*p* = 0.746), indicating that its evolutionary pattern was likely dominated by purifying selection, consistent with the typical of conserved mitochondrial genes (Gissi et al. 2008).

## 4 Conclusion

This study presents the first complete mitochondrial genome sequencing of five representative species within the family Calappidae, revealing their fundamental characteristics (including the typical 37 genes, high AT bias). Phylogenetic analyses combined with divergence time estimation confirmed the monophyly of the genus *Calappa* and indicated that the major diversification of this genus occurred during the Miocene. Selection pressure analyses detected significant positive selection signals in key energy metabolism genes (*ATP6*, *ND2*, *ND5*), providing evidence for understanding the molecular adaptation mechanisms of crustaceans to high‐energy‐demand environments. Furthermore, conserved gene order in Calappidae indicates strong evolutionary constraints, while positive selection in respiratory genes reflects adaptation to hypoxic sediments.

## Author Contributions

**Zhengfei Wang:** conceptualization (equal), data curation (equal), formal analysis (equal), funding acquisition (equal), investigation (equal), methodology (equal), project administration (equal), resources (equal), software (equal), supervision (equal), validation (equal), visualization (equal), writing – original draft (equal), writing – review and editing (equal). **Huiwen Wu:** data curation (equal), formal analysis (equal), investigation (equal), methodology (equal), software (equal), supervision (equal), validation (equal), visualization (equal), writing – original draft (equal), writing – review and editing (equal). **Weijie Jiang:** formal analysis (equal), investigation (equal), methodology (equal), software (equal), supervision (equal), validation (equal), visualization (equal), writing – original draft (equal), writing – review and editing (equal). **Zhiwen Xu:** data curation (equal), investigation (equal), methodology (equal), visualization (equal). **Yuqing Zheng:** data curation (equal), investigation (equal), methodology (equal). **Zhixuan Wang:** formal analysis (equal), software (equal). **Jun Tang:** formal analysis (equal), software (equal). **Xin Chen:** formal analysis (equal).

## Funding

This study was funded by the National Natural Science Foundation of China (grant number 32370436) and the Qinglan project of Jiangsu Province to Wang Zhengfei.

## Ethics Statement

The five species of the genus *Calappa* used in this study (*C. clypeata*, *C. capellonis*, *C. lophos*, *C. hepatica*, and *C. philargius*) are not listed on the International Union for Conservation of Nature Endangered Species List (https://www.iucnredlist.org/). Specimen collection and preservation were performed in strict accordance with the recommendations of the Chinese Guidelines for Quality Assurance in Animal Care. All experimental protocols were approved by the Animal Ethics Committee of Yancheng Teachers University (YCTU221009).

## Conflicts of Interest

The authors declare no conflicts of interest.

## Supporting information


**Figure S1‐1:** Predicted secondary structures of the 22 mitochondrial tRNA genes of *Calappa capellonis*.
**Figure S1‐2:** Predicted secondary structures of the 22 mitochondrial tRNA genes of *Calappa hepatica*.
**Figure S1‐3:** Predicted secondary structures of the 22 mitochondrial tRNA genes of *Calappa clypeata*.
**Figure S1‐4:** Predicted secondary structures of the 22 mitochondrial tRNA genes of *Calappa lophos*.
**Figure S1‐5:** Predicted secondary structures of the 22 mitochondrial tRNA genes of *Calappa philargius*.

**Figure S2:** Structural map of repetitive sequences in the Control Region (CR) of five *Calappa* species.

**Table S1:** The GenBank accession numbers of 189 Brachyura species and 17 Anomura species in this study. The yellow‐highlighted species is the outgroup.

**Table S2:** Fossil calibrations used in divergence time analyses.

**Table S3:** The RSCU analyses of *Calappa lophos*.

**Table S4:** Features of the mitochondrial genome of *Calappa philargius*.

**Table S5:** Composition and skewness of *Calappa philargius* mitogenome.

**Table S6:** CODEML analyses of selection on mitochondrial genes.

**Table S7:** Primer sequences for COX1 and 16SrRNA.

## Data Availability

All the required data are uploaded as Figures S1 and S2, Tables [Link], [Link], [Link], [Link], [Link], [Link], [Link].

## References

[ece373282-bib-0001] Bai, J. , S. Xu , Z. Nie , et al. 2018. “The Complete Mitochondrial Genome of *Huananpotamon lichuanense* (Decapoda: Brachyura) With Phylogenetic Implications for Freshwater Crabs.” Gene 646: 217–226. 10.1016/j.gene.2018.01.015. 29307851

[ece373282-bib-0002] Ballard, J. W. , and M. C. Whitlock . 2004. “The Incomplete Natural History of Mitochondria.” Molecular Ecology 13, no. 4: 729–744. 10.1046/j.1365-294x.2003.02063.x. 15012752

[ece373282-bib-0003] Bellwood, O. 2002. “Systematics, Biogeography and Functional Morphology of the Box Crabs (Family Calappidae).” Research Online, James Cook University. https://www.shurl.cc/de106fed7ee480c12d8bf011830daa1f.

[ece373282-bib-0004] Bernt, M. , C. Bleidorn , A. Braband , et al. 2013. “A Comprehensive Analysis of Bilaterian Mitochondrial Genomes and Phylogeny.” Molecular Phylogenetics and Evolution 69, no. 2: 352–364. 10.1016/j.ympev.2013.05.002. 23684911

[ece373282-bib-0005] Bernt, M. , A. Braband , B. Schierwater , and P. F. Stadler . 2013. “Genetic Aspects of Mitochondrial Genome Evolution.” Molecular Phylogenetics and Evolution 69, no. 2: 328–338. 10.1016/j.ympev.2012.10.020. 23142697

[ece373282-bib-0006] Boore, J. L. 1999. “Animal Mitochondrial Genomes.” Nucleic Acids Research 27, no. 8: 1767–1780. 10.1093/nar/27.8.1767. 10101183 PMC148383

[ece373282-bib-0007] Camargo, T. R. 2018. “Ultrastructure of Spermatozoa of Members of Calappidae, Aethridae and Menippidae and Discussion of Their Phylogenetic Placement.” Acta Zoologica 99: 1–12. 10.1111/azo.12273.

[ece373282-bib-0008] Chen, H. L. 1993. “The Calappidae (Crustacea: Brachyura) of Chinese Waters.” In The Marine Biology of the South China Sea: Proceedings of the First International Conference on the Marine Biology of Hong Kong and the South China Sea, Hong Kong, 28 October–3 November 1990, edited by B. Morton , 675–704. Hong Kong University Press.

[ece373282-bib-0009] De Bruyn, A. , D. P. Martin , and P. Lefeuvre . 2014. “Phylogenetic Reconstruction Methods: An Overview.” Methods in Molecular Biology 1115: 257–277. 10.1007/978-1-62703-767-9_13. 24415479

[ece373282-bib-0010] Dhar, D. , D. Dey , S. Basu , and H. Fortunato . 2021. “Understanding the Adaptive Evolution of Mitochondrial Genomes in Intertidal Chitons.” Journal of Molluscan Studies 87, no. 2: eyab018. 10.1093/mollus/eyab018.

[ece373282-bib-0011] Drummond, A. J. , and A. Rambaut . 2007. “BEAST: Bayesian Evolutionary Analysis by Sampling Trees.” BMC Evolutionary Biology 7: 214. 10.1186/1471-2148-7-214. 17996036 PMC2247476

[ece373282-bib-0012] Edgar, R. C. 2004. “MUSCLE: Multiple Sequence Alignment With High Accuracy and High Throughput.” Nucleic Acids Research 32, no. 5: 1792–1797. 10.1093/nar/gkh340. 15034147 PMC390337

[ece373282-bib-0013] Ewers‐Saucedo, C. , J. P. Wares , R. Hanel , and D. Brandis . 2016. “Evolution of Male Copulatory Organs in Box Crabs (Decapoda: Eubrachyura: Calappidae de Haan, 1833).” Journal of Crustacean Biology 36, no. 6: 804–814.

[ece373282-bib-0014] Galil, B. S. 1997. “Crustacea Decapoda: A Revision of the Indo‐Pacific Species of the Genus *Calappa* Weber, 1795 (Calappidae).” In Résultats Des Campagnes MUSORSTOM 18. Mémoires du Muséum national d'Histoire naturelle. Série A, Zoologie 176, edited by A. Crosnier , 271–335. Éditions du Muséum.

[ece373282-bib-0015] Galil, B. S. , and P. F. Clark . 1994. “A Revision of the Genus *Matuta* Weber, 1795 (Crustacea: Brachyura: Calappidae).” Zoologische Verhandelingen 294: 1–55.

[ece373282-bib-0016] Gissi, C. , F. Iannelli , and G. Pesole . 2008. “Evolution of the Mitochondrial Genome of Metazoa as Exemplified by Comparison of Congeneric Species.” Heredity 101, no. 4: 301–320. 10.1038/hdy.2008.62. 18612321

[ece373282-bib-0017] Guinot, D. , M. Tavares , and P. Castro . 2013. “Significance of the Sexual Openings and Supplementary Structures on the Phylogeny of Brachyuran Crabs (Crustacea, Decapoda, Brachyura), With New Nomina for Higher‐Ranked Podotreme Taxa.” Zootaxa 3665: 1–414. 10.11646/zootaxa.3665.1.1. 26401537

[ece373282-bib-0018] Hassanin, A. , N. Leger , and J. Deutsch . 2005. “Evidence for Multiple Reversals of Asymmetric Mutational Constraints During the Evolution of the Mitochondrial Genome of Metazoa, and Consequences for Phylogenetic Inferences.” Systematic Biology 54, no. 2: 277–298. 10.1080/10635150590947843. 16021696

[ece373282-bib-0019] Huelsenbeck, J. P. , and F. Ronquist . 2001. “MRBAYES: Bayesian Inference of Phylogenetic Trees.” Bioinformatics 17, no. 8: 754–755. 10.1093/bioinformatics/17.8.754. 11524383

[ece373282-bib-0020] Kosakovsky, P. S. , B. Murrell , M. Fourment , S. D. Frost , W. Delport , and K. Scheffler . 2011. “A Random Effects Branch‐Site Model for Detecting Episodic Diversifying Selection.” Molecular Biology and Evolution 28, no. 11: 3033–3043. 10.1093/molbev/msr125. 21670087 PMC3247808

[ece373282-bib-0021] Li, G. , Y. Zheng , X. Mao , et al. 2016. “Sequencing and Analysis of the Complete Mitochondrial Genome of *Eriocheir sinensis* .” Mitochondrial DNA Part A DNA Mapping, Sequencing, and Analysis 27, no. 6: 4039–4040. 10.3109/19401736.2014.1003833. 25629479

[ece373282-bib-0022] Liu, Y. , Y. Liao , J. Lv , Y. Li , and R. Liu . 2022. “Characterization of the Complete Mitochondrial Genome of the Bromeliad Crab *Metopaulias depressus* (Rathbun, 1896) (Crustacea: Decapoda: Brachyura: Sesarmidae).” Genes 13, no. 2: 299. 10.3390/genes13020299. 35205344 PMC8872168

[ece373282-bib-0023] Luque, J. , H. D. Bracken‐Grissom , J. Ortega‐Hernández , and J. M. Wolfe . 2024. “Fossil Calibrations for Molecular Analyses and Divergence Time Estimation for True Crabs (Decapoda: Brachyura).” Palaeontologia Electronica 27, no. 2: a38. 10.26879/1332.

[ece373282-bib-0024] Ma, K. Y. , J. Qin , C. W. Lin , et al. 2019. “Phylogenomic Analyses of Brachyuran Crabs Support Early Divergence of Primary Freshwater Crabs.” Molecular Phylogenetics and Evolution 135: 62–66. 10.1016/j.ympev.2019.02.001. 30763757

[ece373282-bib-0025] Marin, I. N. , and A. V. Tiunov . 2023. “Terrestrial Crustaceans (Arthropoda, Crustacea): Taxonomic Diversity, Terrestrial Adaptations, and Ecological Functions.” ZooKeys 1169: 95–162. 10.3897/zookeys.1169.97812. 38328027 PMC10848873

[ece373282-bib-0026] McGaw, I. J. 2005. “Burying Behaviour of Two Sympatric Crab Species: Cancer Magister and Cancer Productus.” Scientia Marina 69: 375–381. 10.3989/scimar.2005.69n3375.

[ece373282-bib-0027] Nguyen, L. T. , H. A. Schmidt , A. von Haeseler , and B. Q. Minh . 2015. “IQ‐TREE: A Fast and Effective Stochastic Algorithm for Estimating Maximum‐Likelihood Phylogenies.” Molecular Biology and Evolution 32, no. 1: 268–274. 10.1093/molbev/msu300. 25371430 PMC4271533

[ece373282-bib-0028] Pan, D. , Y. Sun , B. Shi , et al. 2024. “Phylogenomic Analysis of Brachyuran Crabs Using Transcriptome Data Reveals Possible Sources of Conflicting Phylogenetic Relationships Within the Group.” Molecular Phylogenetics and Evolution 201: 108201. 10.1016/j.ympev.2024.108201. 39278384

[ece373282-bib-0029] Rambaut, A. , A. J. Drummond , D. Xie , G. Baele , and M. A. Suchard . 2018. “Posterior Summarization in Bayesian Phylogenetics Using Tracer 1.7.” Systematic Biology 67, no. 5: 901–904. 10.1093/sysbio/syy032. 29718447 PMC6101584

[ece373282-bib-0030] Ruan, H. , M. Li , Z. Li , et al. 2020. “Comparative Analysis of Complete Mitochondrial Genomes of Three *Gerres* Fishes (Perciformes: Gerreidae) and Primary Exploration of Their Evolution History.” International Journal of Molecular Sciences 21, no. 5: 1874. 10.3390/ijms21051874. 32182936 PMC7084342

[ece373282-bib-0031] Saccone, C. , C. De Giorgi , C. Gissi , G. Pesole , and A. Reyes . 1999. “Evolutionary Genomics in Metazoa: The Mitochondrial DNA as a Model System.” Gene 238, no. 1: 195–209. 10.1016/s0378-1119(99)00270-x. 10570997

[ece373282-bib-0032] Saito, S. , K. Tamura , and T. Aotsuka . 2005. “Replication Origin of Mitochondrial DNA in Insects.” Genetics 171, no. 4: 1695–1705. 10.1534/genetics.105.046243. 16118189 PMC1456096

[ece373282-bib-0033] Sanger, F. , and A. R. Coulson . 1975. “A Rapid Method for Determining Sequences in DNA by Primed Synthesis With DNA Polymerase.” Journal of Molecular Biology 94, no. 3: 441–448. 10.1016/0022-2836(75)90213-2. 1100841

[ece373282-bib-0034] Sbisa, E. , F. Tanzariello , A. Reyes , G. Pesole , and C. Saccone . 1997. “Mammalian Mitochondrial d‐Loop Region Structural Analysis: Identification of New Conserved Sequences and Their Functional and Evolutionary Implications.” Gene 205, no. 1–2: 125–140. 10.1016/s0378-1119(97)00404-6. 9461386

[ece373282-bib-0035] Schneider, A. 2011. “Mitochondrial tRNA Import and Its Consequences for Mitochondrial Translation.” Annual Review of Biochemistry 80: 1033–1053. 10.1146/annurev-biochem-060109-092838. 21417719

[ece373282-bib-0036] Seed, R. , and R. N. Hughes . 1995. “Criteria for Prey Size‐Selection in Molluscivorous Crabs With Contrasting Claw Morphologies.” Journal of Experimental Marine Biology and Ecology 193: 177–195. 10.1016/0022-0981(95)00117-4.

[ece373282-bib-0037] Simon, C. , T. R. Buckley , F. Frati , and A. T. Beckenbach . 2006. “Incorporating Molecular Evolution Into Phylogenetic Analysis, and a New Compilation of Conserved Polymerase Chain Reaction Primers for Animal Mitochondrial DNA.” Annual Review of Ecology, Evolution, and Systematics 37: 545–579. 10.1146/annurev.ecolsys.37.091305.110018.

[ece373282-bib-0038] Sung, J. M. , J. Lee , S. G. Kim , M. Z. Karagozlu , and C. B. Kim . 2016. “Analysis of Complete Mitochondrial Genome of *Ocypode cordimanus* (Latreille, 1818) (Decapoda, Ocypodidae).” Mitochondrial DNA Part B‐Resources 1, no. 1: 363–364. 10.1080/23802359.2016.1168718. 33490393 PMC7800992

[ece373282-bib-0039] Tan, M. H. , H. M. Gan , Y. P. Lee , et al. 2018. “ORDER Within the Chaos: Insights Into Phylogenetic Relationships Within the Anomura (Crustacea: Decapoda) From Mitochondrial Sequences and Gene Order Rearrangements.” Molecular Phylogenetics and Evolution 127: 320–331. 10.1016/j.ympev.2018.05.015. 29800651

[ece373282-bib-0040] Tsang, C. T. T. , C. D. Schubart , K. H. Chu , P. K. L. Ng , and L. M. Tsang . 2022. “Molecular Phylogeny of Thoracotremata Crabs (Decapoda, Brachyura): Toward Adopting Monophyletic Superfamilies, Invasion History Into Terrestrial Habitats and Multiple Origins of Symbiosis.” Molecular Phylogenetics and Evolution 177: 107596. 10.1016/j.ympev.2022.107596. 35914646

[ece373282-bib-0041] Tsang, L. M. , C. D. Schubart , S. T. Ahyong , et al. 2014. “Evolutionary History of True Crabs (Crustacea: Decapoda: Brachyura) and the Origin of Freshwater Crabs.” Molecular Biology and Evolution 31, no. 5: 1173–1187. 10.1093/molbev/msu068. 24520090

[ece373282-bib-0042] Wang, Q. , D. Tang , H. Guo , J. Wang , X. Xu , and Z. Wang . 2020. “Comparative Mitochondrial Genomic Analysis of Macrophthalmus Pacificus and Insights Into the Phylogeny of the Ocypodoidea & Grapsoidea.” Genomics 112, no. 1: 82–91. 10.1016/j.ygeno.2019.12.012. 31863840

[ece373282-bib-0043] Wang, Q. , Z. Wang , D. Tang , et al. 2020. “Characterization and Comparison of the Mitochondrial Genomes From Two Alpheidae Species and Insights Into the Phylogeny of Caridea.” Genomics 112, no. 1: 65–70. 10.1016/j.ygeno.2019.08.013. 31437541

[ece373282-bib-0044] Wang, Z. , C. Ji , Z. Wang , et al. 2018. “The Complete Mitogenome of *Metopograpsus quadridentatus* and Phylogenetic Analysis.” Mitochondrial DNA Part B‐Resources 3, no. 2: 1169–1171. 10.1080/23802359.2018.1524272. 33490568 PMC7800309

[ece373282-bib-0045] Welch, J. J. , A. Eyre‐Walker , and D. Waxman . 2008. “Divergence and Polymorphism Under the Nearly Neutral Theory of Molecular Evolution.” Journal of Molecular Evolution 67, no. 4: 418–426. 10.1007/s00239-008-9146-9. 18818860

[ece373282-bib-0046] Wolfe, J. M. , L. Ballou , J. Luque , et al. 2024. “Convergent Adaptation of True Crabs (Decapoda: Brachyura) to a Gradient of Terrestrial Environments.” Systematic Biology 73, no. 2: 247–262. 10.1093/sysbio/syad066. 37941464 PMC11282366

[ece373282-bib-0047] Wolfe, J. M. , J. W. Breinholt , K. A. Crandall , et al. 2019. “A Phylogenomic Framework, Evolutionary Timeline and Genomic Resources for Comparative Studies of Decapod Crustaceans.” Proceedings of the Royal Society B‐Biological Sciences 286, no. 1901: 20190079. 10.1098/rspb.2019.0079. PMC6501934 31014217

[ece373282-bib-0048] Yang, M. , D. Dong , and X. Li . 2021. “The Complete Mitogenome of *Phymorhynchus* sp. (Neogastropoda, Conoidea, Raphitomidae) Provides Insights Into the Deep‐Sea Adaptive Evolution of Conoidea.” Ecology and Evolution 11, no. 12: 7518–7531. 10.1002/ece3.7582. 34188831 PMC8216942

[ece373282-bib-0049] Yang, M. , L. Gong , J. Sui , and X. Li . 2019. “The Complete Mitochondrial Genome of *Calyptogena marissinica* (Heterodonta: Veneroida: Vesicomyidae): Insight Into the Deep‐Sea Adaptive Evolution of Vesicomyids.” PLoS One 14, no. 9: e0217952. 10.1371/journal.pone.0217952. 31536521 PMC6752807

[ece373282-bib-0050] Yang, Z. , and R. Nielsen . 2000. “Estimating Synonymous and Nonsynonymous Substitution Rates Under Realistic Evolutionary Models.” Molecular Biology and Evolution 17, no. 1: 32–43. 10.1093/oxfordjournals.molbev.a026236. 10666704

[ece373282-bib-0051] Zhang, D. X. , and G. M. Hewitt . 1996. “Nuclear Integrations: Challenges for Mitochondrial DNA Markers.” Trends in Ecology & Evolution 11, no. 6: 247–251. 10.1016/0169-5347(96)10031-8. 21237827

[ece373282-bib-0052] Zhang, Y. , L. Wei , B. Liu , L. Liu , Z. Lü , and L. Gong . 2023. “Two Complete Mitogenomes of Ocypodoidea (Decapoda: Brachyura), *Cleistostoma dilatatum* (Camptandriidae) and *Euplax* sp. (Macrophthalmidae) and Its Phylogenetic Implications.” Acta Oceanologica Sinica 42, no. 4: 81–92. 10.1007/s13131-022-2054-9.

