# Supplementary material for: Conserved Gene Order and Adaptive Evolution in Mitochondrial Genomes of Calappa Crabs: Insights Into Ecological Specialization and Phylogenetic Utility
Source: Ecol Evol. 2026 Mar 20;16(3):e73282. doi: 10.1002/ece3.73282 (PMC13093676; doi:10.1002/ece3.73282)
Supplement: Supplementary file 1 — Figure S1‐1: Predicted secondary structures of the 22 mitochondrial tRNA genes of Calappa capellonis. Figure S1‐2: Predicted secondary structures of the 22 mitochondrial tRNA genes of Calappa hepatica. Figure S1‐3: Predicted secondary structures of the 22 mitochondrial tRNA genes of Calappa clypeata. Figure S1‐4: Predicted secondary structures of the 22 mitochondrial tRNA genes of Calappa lophos. Figure S1‐5: Predicted secondary structures of the 22 mitochondrial tRNA genes of Calappa philargius. [file ECE3-16-e73282-s006.pdf]

Calappa capellonis

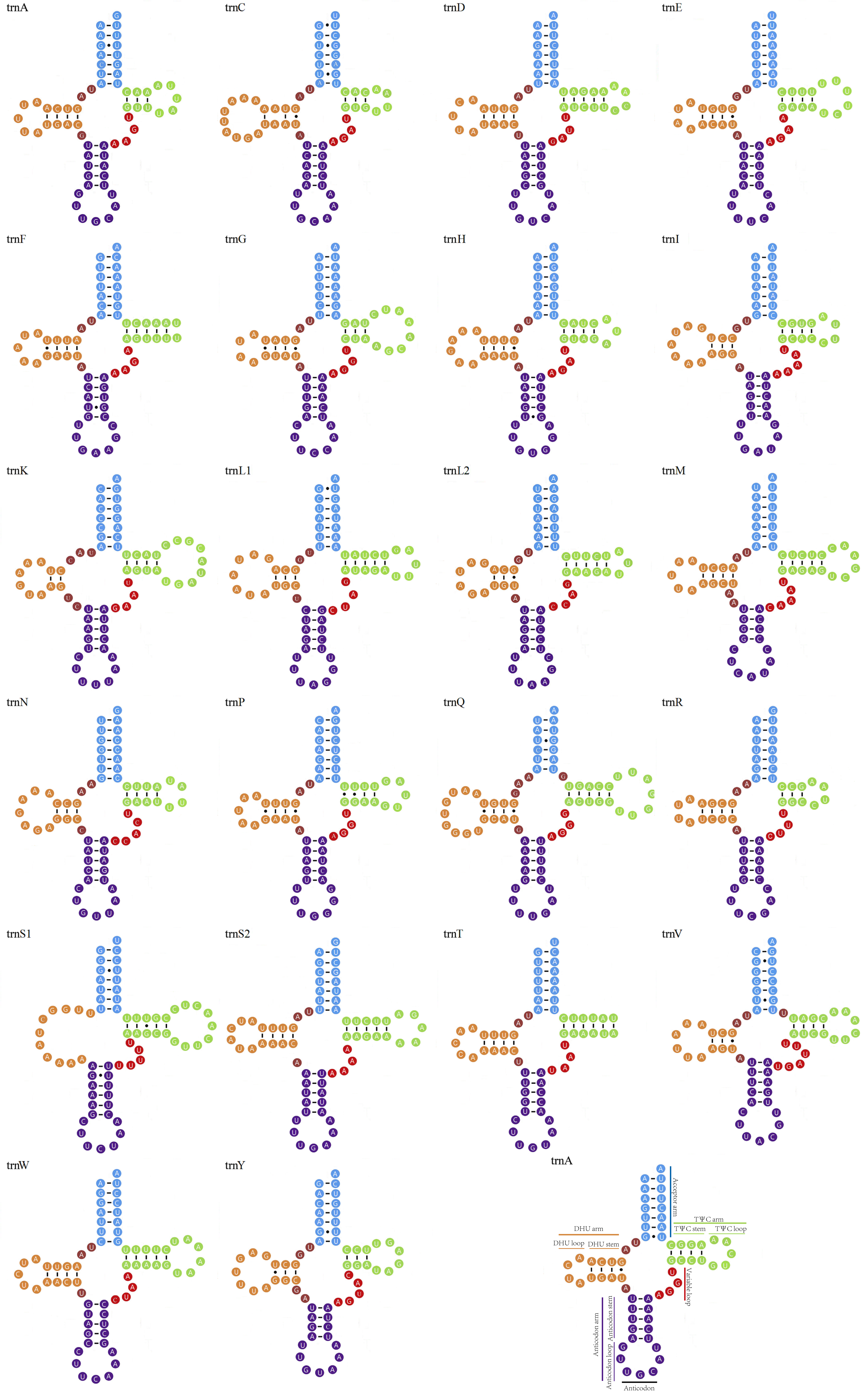

Figure S1-1. Predicted secondary structures of the 22 mitochondrial tRNA genes of *Calappa capellonis.*

Calappa hepatica

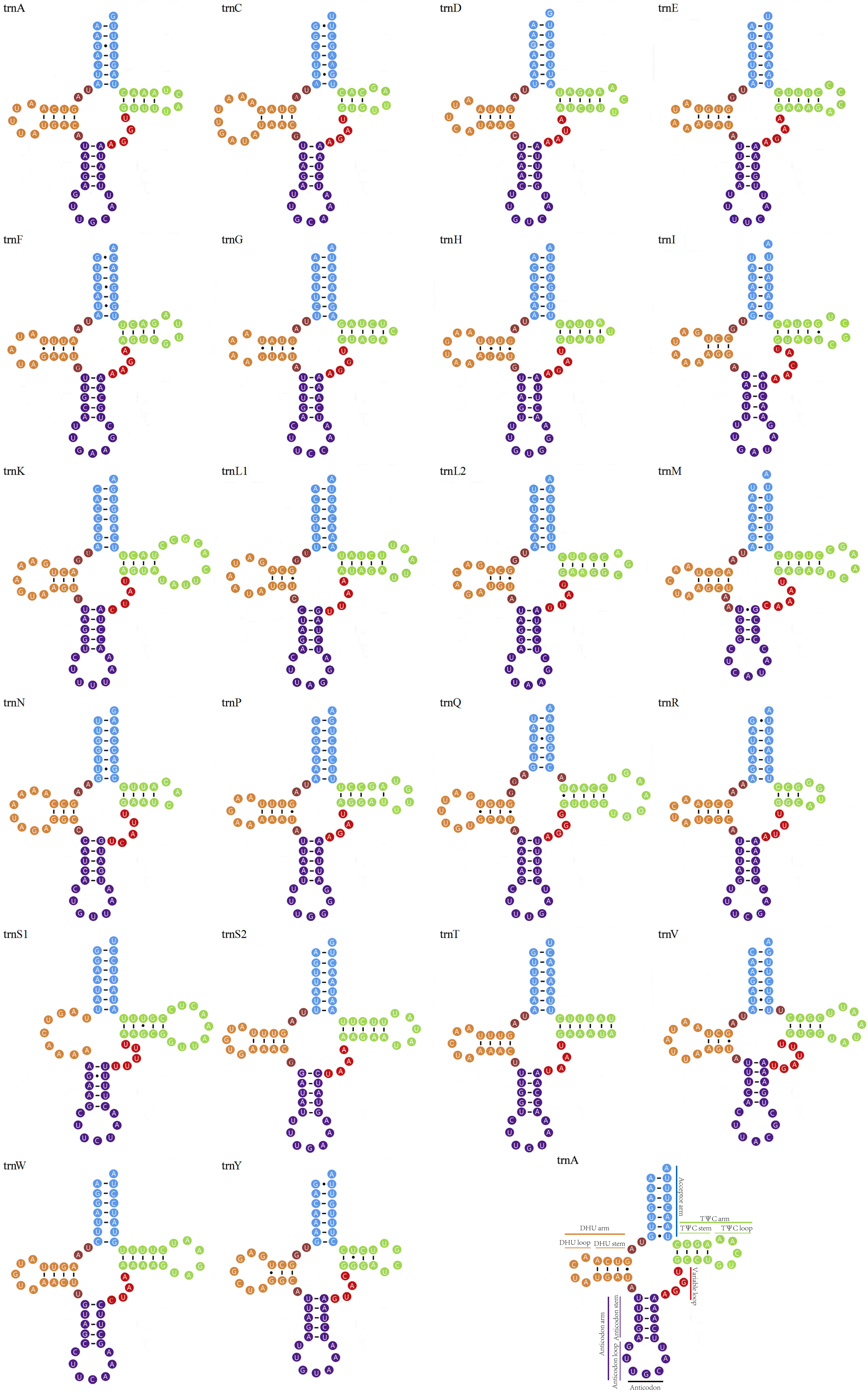

Figure S1-2. Predicted secondary structures of the 22 mitochondrial tRNA genes of *Calappa hepatica.*

Calappa clypeata

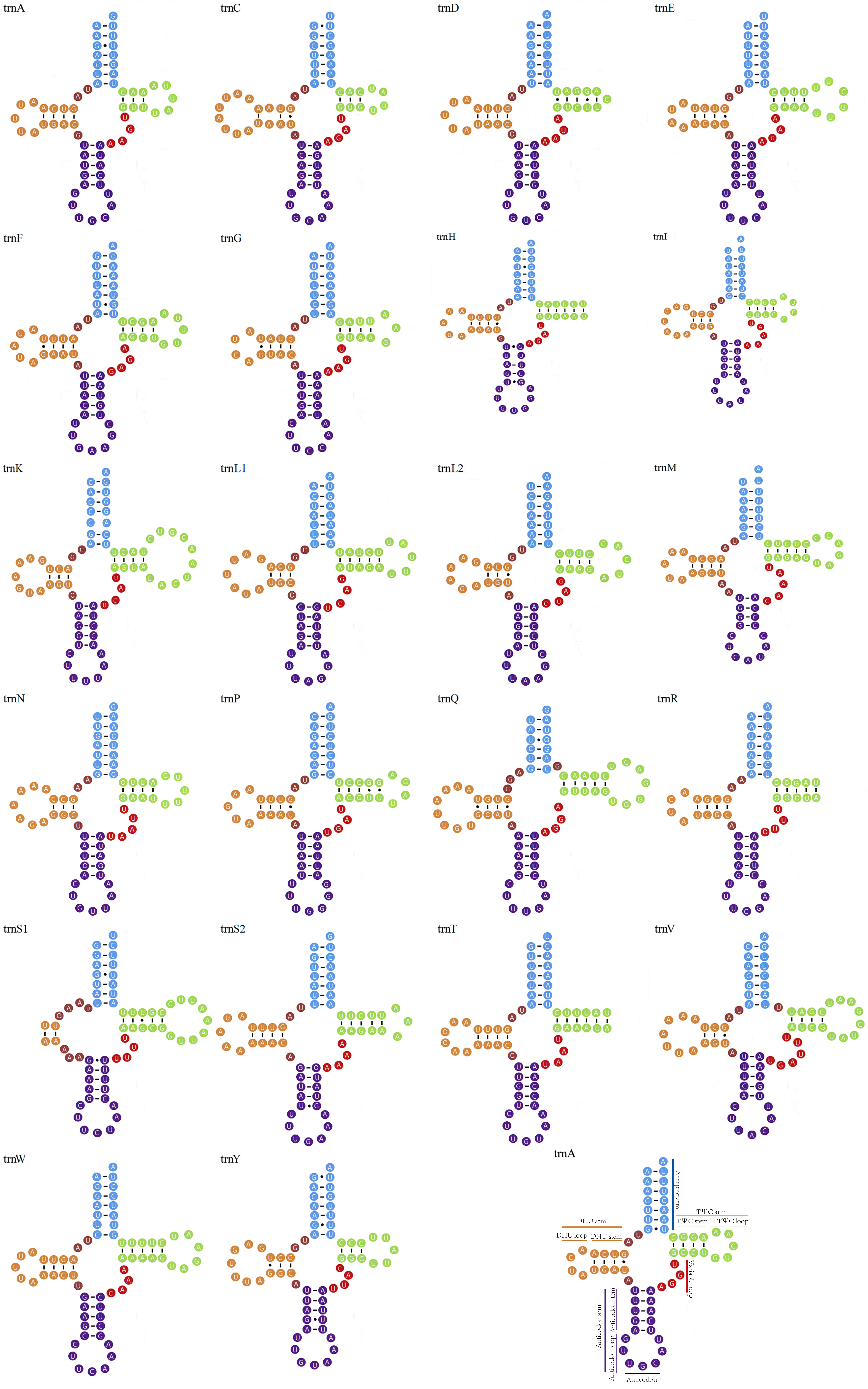

Figure S1-3. Predicted secondary structures of the 22 mitochondrial tRNA genes of *Calappa clypeata.*

Calappa lophos

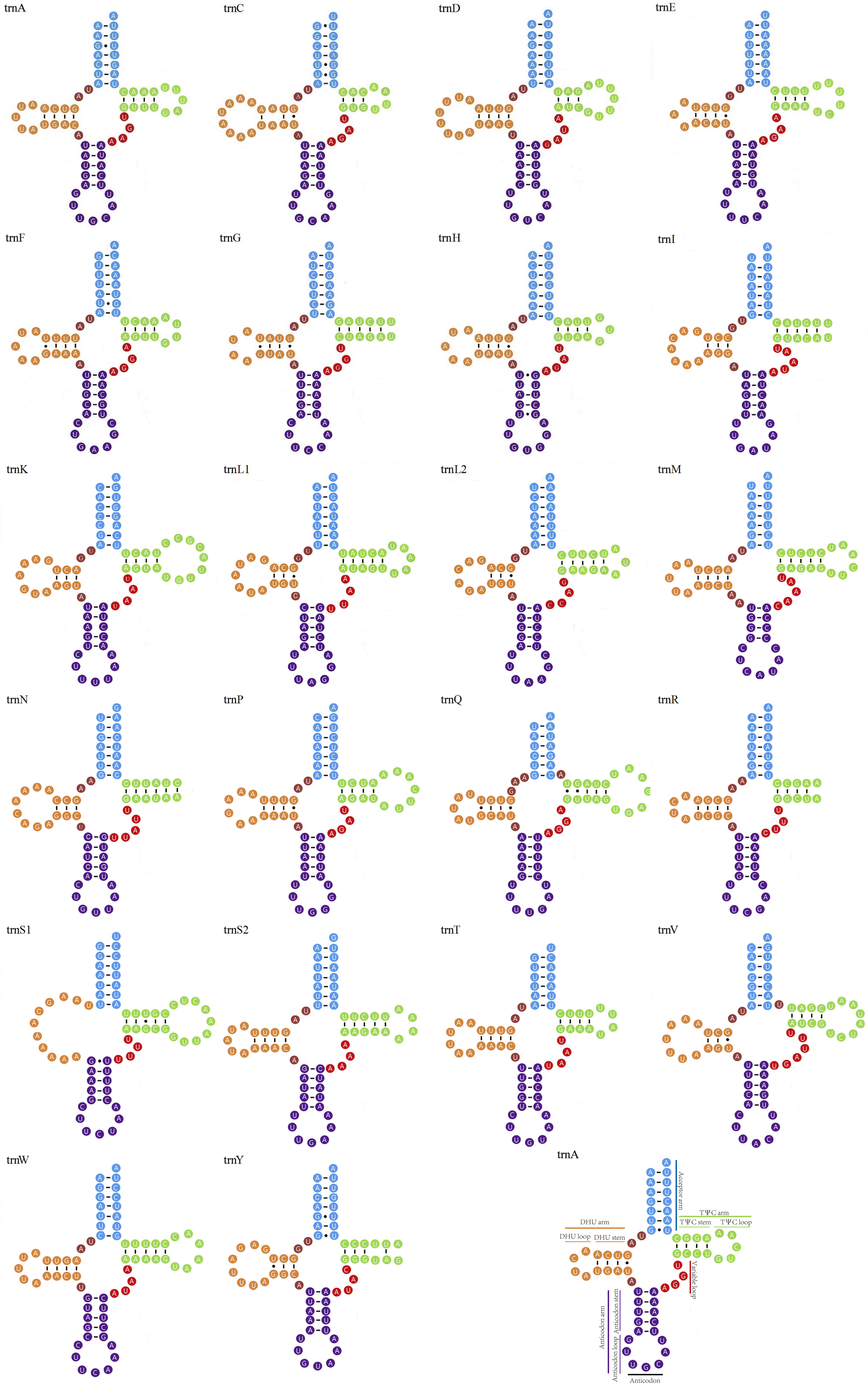

Figure S1-4. Predicted secondary structures of the 22 mitochondrial tRNA genes of *Calappa lophos*.

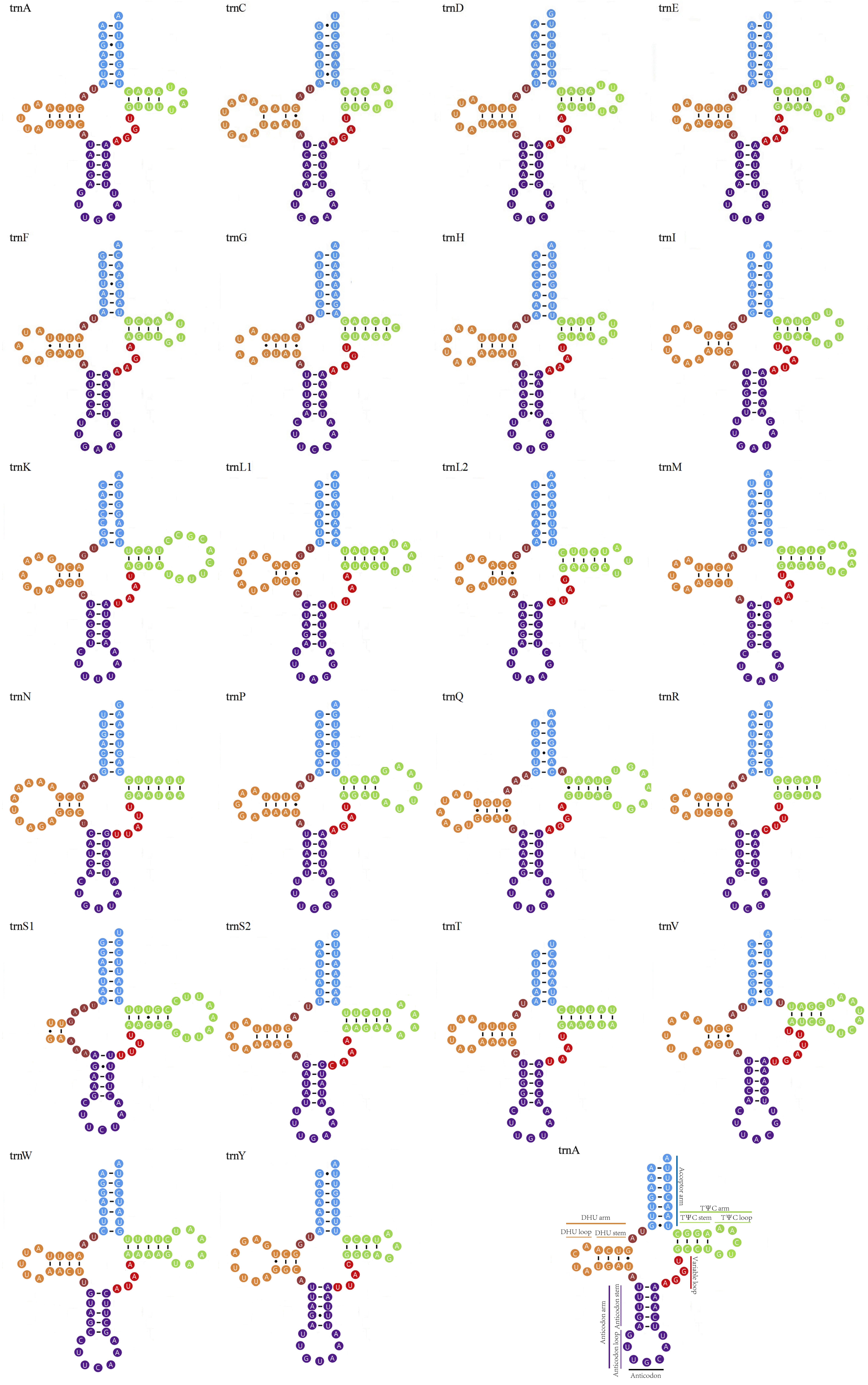

Figure S1-5. Predicted secondary structures of the 22 mitochondrial tRNA genes of *Calappa philargius.*
