# Supplementary material for: Conserved Gene Order and Adaptive Evolution in Mitochondrial Genomes of Calappa Crabs: Insights Into Ecological Specialization and Phylogenetic Utility
Source: Ecol Evol. 2026 Mar 20;16(3):e73282. doi: 10.1002/ece3.73282 (PMC13093676; doi:10.1002/ece3.73282)
Supplement: Supplementary file 2 — Figure S2: Structural map of repetitive sequences in the Control Region (CR) of five Calappa species. [file ECE3-16-e73282-s008.pdf]

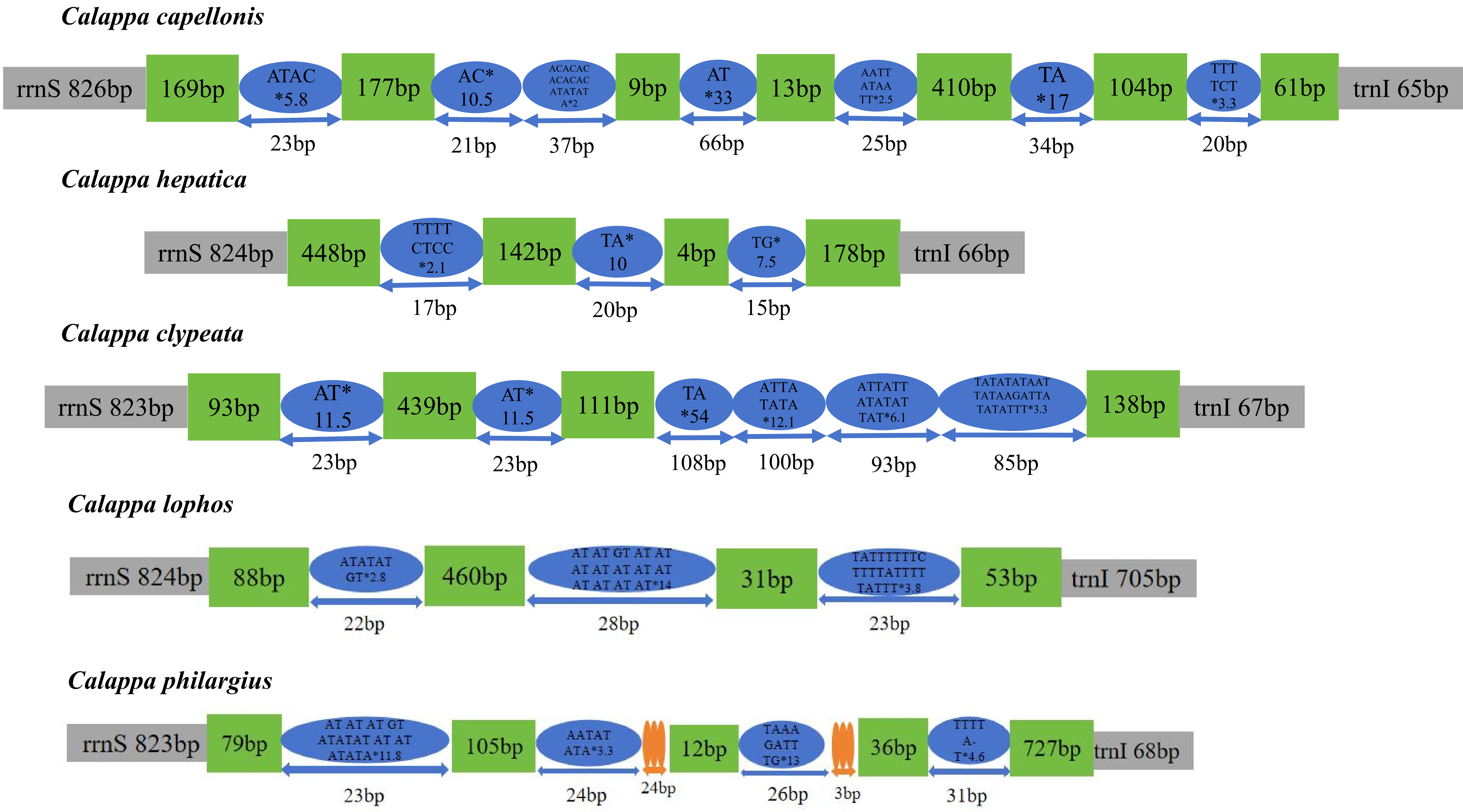

Figure S2. Structural map of repetitive sequences in the Control Region (CR) of five *Calappa* species.
